# Metal grafted graphene-based nanomaterials towards cancer theranostic efficacy

**DOI:** 10.37349/etat.2025.1002346

**Published:** 2025-11-10

**Authors:** Prashant H. Gohil, Gopal Avashthi

**Affiliations:** University of Valladolid, Spain; School of Sciences, P P Savani University, Dhamdod, Kosamba, Surat 394125, Gujarat, India

**Keywords:** metal-grafted graphene nanohybrids, cancer theranostics, targeted drug delivery, reactive oxygen species (ROS)

## Abstract

Cancer is one of the leading global causes of mortality and morbidity, so it needs early diagnosis and therapies. Traditional diagnostic and therapeutic strategies are inadequate due to several limitations, such as poor specificity, systemic toxicity, and delays, while metal-grafted Gr nanostructures have emerged as promising theranostic platforms due to their unique electronic, optical, and structural properties. Metals such as Fe_3_O_4_, Au, Ag, TiO_2_, Pd, Pt, Bi, ZnO, and Cu grafted onto the Gr surface impart electronic modulation, enhance surface area, flexibility, conductivity, reactivity, biomolecular interactions, and biosensing, thereby enabling precise biomarker detection, targeted drug delivery, imaging, and photothermal/photodynamic therapy (PTT/PDT). Eco-friendly synthesis using plant extracts and microbes offers a sustainable and biocompatible alternative to conventional chemical synthesis. However, challenges remain, such as homogenous doping, synthetic complexity, long-term safety, and clinical scalability. Innovations such as scalable, cost-effective, biocompatible nanofibers, nanopapers, microfluidic, and wearable biosensors are being explored by incorporating AI and advanced diagnostic tools for advanced biomedical devices. In vitro, half maximum inhibitory concentrations (IC_50_) studies show that size- and dose-dependent nanohybrids such as Fe_3_O_4_-Gr, γ-Fe_2_O_3_-Gr, Au-Gr, and Bi-Gr exhibited safer responses at lower concentrations 10–200 µg/mL across HBE, MCF-7, HeLa B, and LNCaP cell lines. Bi-Gr was tested on human liver cancer (HepG2) cell line, which exhibits higher reactivity despite a safer profile of Bi at ~53–88 µg/mL. Pd-Gr and Pt-Gr significantly reduced viability in prostate and ovarian cancer cells at 10–50 µg/mL, while ZnO-Gr, Ag-Gr, and Cu-Gr showed safer activity at lower concentrations on MCF-7. In vivo studies remain limited; median lethal dose (LD_50_) values for Fe_3_O_4_-Gr and γ-Fe_2_O_3_-Gr were determined to be associated with rapid lethal biodistribution observed in the liver, lungs, and spleen. Metal-grafted Gr nanohybrids demonstrate immense potential for multifunctional cancer theranostics, though systematic in vivo toxicity studies still need to be explored by the intravenously administered route to lower the LD_50_ of nanohybrids for their clinical translation.

## Graphical abstract

**Graphical abstract.**
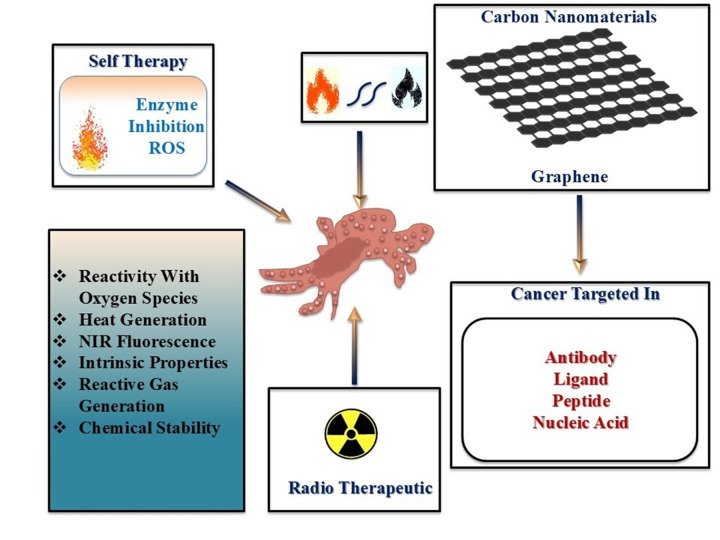
** Multifaceted graphene-based nanomaterials for cancer therapy.** ROS: reactive oxygen species; NIR: near-infrared.

## Introduction

**Figure 1:**
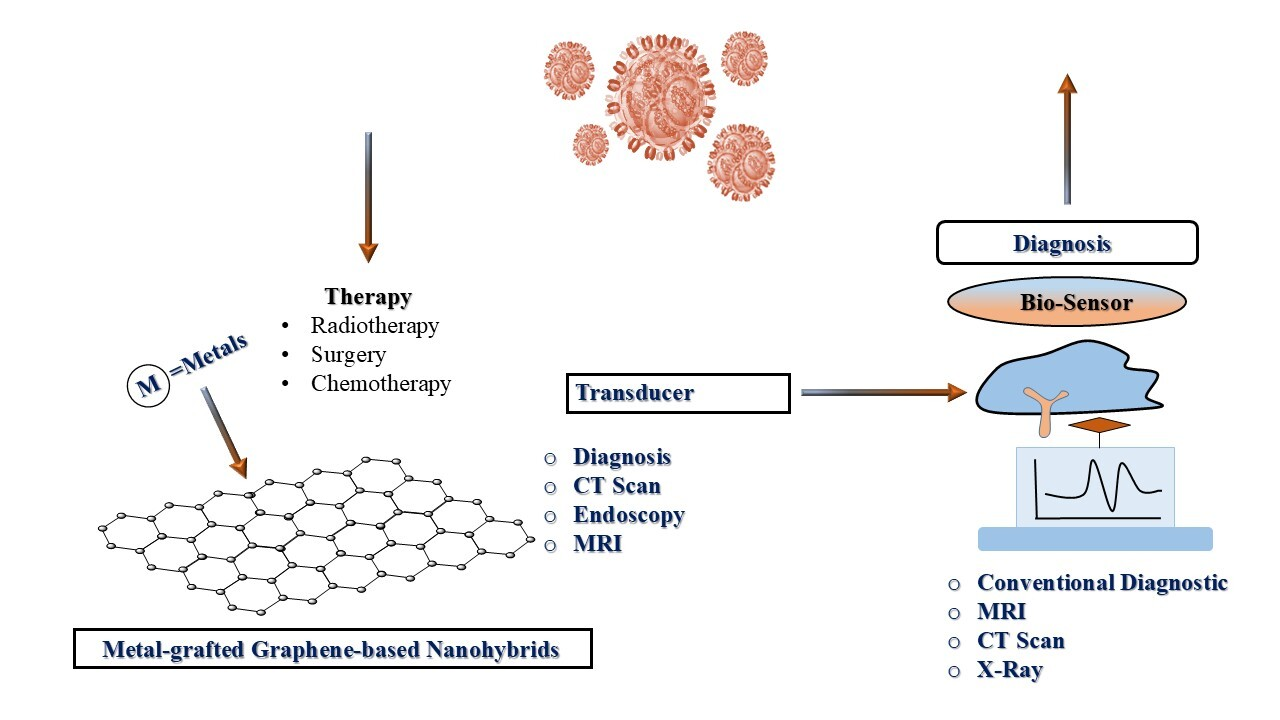
**Graphene nanohybrids as multifunctional platforms for cancer diagnosis and therapy.** MRI: magnetic resonance imaging.

Cancer is a multifaceted disease characterized by the uncontrolled growth of abnormal cells [1]. WHO projected global cancer incidence statistics of 29.5 million new cases and 16.4 million deaths by 2040 [2]. The three most common cancers worldwide are breast, lung, and prostate cancers. Early diagnosis is crucial for improving treatment efficacy and patient survival [3, 4]. Conventional diagnostic methods, including X-rays, mammography, magnetic resonance imaging (MRI), CT scans, endoscopy, and Pap smears, are commonly employed but suffer from notable drawbacks such as lengthy procedures and high expenses. So, there is a need for specialized expertise, particularly in preventive early-stage cancer detection [5–8] (Figure 1). The discussions are focused notably on designing, properties, advantages, disadvantages, applications, and toxicity study of carbon-based materials like graphene oxide (GO), reduced GO (rGO), Gr quantum dots, carbon nanotubes (CNTs), and metal-grafted Gr-based nanomaterials for improving the cancer theranostics. Size and time-dependent cell viability studies of Au-Gr, Fe_3_O_4_-Gr, ZnO-Gr, Pd-Gr, Pt-Gr, Bi-Gr, Cu-Gr, and Ag-Gr, along with their mechanisms in cancer treatment, have strengthened the study. Au-Gr composites improve photothermal therapy (PTT) by converting near-infrared (NIR) light into heat to kill cancerous cells, while Fe_3_O_4_-Gr nanohybrids provide MRI and magnetically guided drug delivery. Additional systems, such as rGO-TiO_2_, enable efficient reactive oxygen species (ROS) production for photodynamic therapy (PDT). Grafting several metals onto the Gr surface suggests multifunctional platforms for integrated cancer diagnosis and treatment simultaneously. Also, complex and scalable structural synthesis may create complications for achieving homogenous doped nanohybrids. However, their toxicity results at the laboratory scale may not be directly translated into clinical outcomes. Study highlights the rapidly growing interest of researchers in the field of Gr and its uses for cancer therapy, focusing on its primary applications in drug delivery, optical photothermal (PT) and photodynamic (PD) theranostics efficacy, which help to treat cancerous cells with real-time diagnosis [9]. However, the Gr-based materials have numerous potentials; several drawbacks persist. Its poor solubility, dispersibility, and low bioavailability in physiological media, unpredictable metabolization and drug elimination from the body, non-specific selectivity, and side effects on normal tissues are drawbacks of these composites. These fundamental issues hinder its uniform distribution throughout the body and compromise its stability and presenting a key challenge to its successful clinical application. Moreover, there is a need to reframe the discussion of challenges, including cytotoxicity, synthesis scalability, and barriers to clinical translation. So, this ensures that the reported argument for the continued investigation of metal-grafted graphene-based nanomaterials is vibrant, compelling, and aligned with current scientific discourse. Improved performance of graphene-based nanocomposites is directly associated with their synergistic physicochemical properties, such as high surface area, excellent electron mobility, and inherent biocompatibility, which make it highly suitable for drug delivery and biosensing for efficient electrochemical sensors, catalytic, and therapeutic applications. V_3.6_Mo_2.6_O_16_-chitosan (Mv-CHT) nanostructure provides abundant electroactive sites and high electron mobility, in which CHT ensures colloidal stability and biocompatibility, thereby lowering the detection limit to the nanomolar range [10]. Similarly, the anti-cancer efficacy of NiCO_2_O_4_/NiO nanoparticles (NPs) is primarily attributed to their ability to generate ROS, which induce mitochondrial dysfunction and apoptosis in breast cancer cells [11]. Cobalt oxide (CO_3_O_4_) NPs, on the other hand, act as catalyzers in the Fenton-like reaction, accelerating the decomposition of H_2_O_2_ into (^•^OH) for improving tumor cell killing efficacy. The factors such as particle size, surface charge, doping consistency, and reproducibility influence biodistribution and cellular uptake through eco-friendly synthesis. Synergistic effects of metals-graphene interactions and their comparative analysis of systems (Au-Gr, Fe_3_O_4_-Gr, ZnO-Gr, Pd-Gr, Pt-Gr, Bi-Gr, Cu-Gr, and Ag-Gr) have been included to contextualize clinical suitability.

Alternatively, biosensors have emerged as innovative diagnostic tools capable of accurately identifying cancer biomarkers. It incorporates a bio-recognition element that specifically interacts with a target biomarker and a transducer that is responsible for biological interaction as a detectable signal. Successful early diagnosis often depends on detecting tumor-specific biomarkers, including peptides, enzymes, proteins, nucleic acids, and antibodies, present in biological fluids like blood, urine, or sweat [12–16]. Their advantages include low cost, rapid response, sensitivity, and minimal sample requirements. Cancer treatment strategies include chemotherapy, radiotherapy, surgical interventions, or multimodal therapies [17–19]. Radiotherapy utilizes ionizing radiation to induce DNA damage in cancer cells and is delivered externally through external beam radiotherapy (EBRT) or internally via brachytherapy and radionuclide injections [20, 21]. Although EBRT is widely used and effective, it lacks specificity and often affects surrounding healthy tissues. Brachytherapy, on the other hand, provides more localized treatment but depends on precise tumor targeting [22]. Despite being a fundamental component of cancer treatment and the non-selective nature of ionizing radiation poses a risk of collateral damage to normal cells. A cornerstone of ionizing radiation of cancer therapy primarily exerts its effects by generating ROS, which induce DNA damage such as single-strand breaks (SSBs) and, more critically, double-strand breaks (DSBs) that can lead to apoptosis if unrepaired [23–25]. However, its non-specific mechanism of action often results in collateral damage to healthy tissues. EBRT delivers radiation from an external source that directly affects both cancerous and surrounding normal cells, so this highlights the need for more targeted approaches. Despite advances in medical treatments, cancer remains the second leading cause of death globally, accounting for one in six deaths, with more than 70% of fatalities occurring in low- and middle-income countries. Risk factors include tobacco use, obesity, unhealthy diets, sedentary lifestyles, and alcohol consumption. The complexity of cancer is further amplified by its genetic heterogeneity, multidrug resistance, and tendency for recurrence. Its development is driven by numerous mutations often affecting tumor suppressor genes such as p53, which normally plays a crucial role in regulating apoptosis [26, 27]. Malignant cells often evade apoptotic mechanisms and proliferate rapidly, infiltrating surrounding tissues. Due to the diversity of genetic mutations, pinpointing a single causative mutation per cancer type is exceedingly difficult. Biosensors are classified based on their bio-receptor type: catalytic biosensors employ enzymes, while biosensors with affinity use antibodies, nucleic acids, or proteins [28, 29]. Used transducers in these devices act through optical, electrochemical, piezoelectric, or calorimetric mechanisms [30–32]. Enhancing biosensor performance involves metal-grafting using precursors such as Au-TiO_2_, Ag-ZnO, Fe_3_O_4_, CuO, CeO_2_, MnO_2_, and MoS_2_. It is investigated that several factors like synergistic mechanism, morphology control, phase transformations, and surface functionalization, show a critical role for enhancing biosensor performance. Hydrothermally engineered Cu_5_V_2_O_10_ nanostructures delivered ultra-low detection limits for mefenamic acid due to optimized crystallinity and high surface area [33]. Such a method has been applied first time to synthesize such nanomaterials, which enables accurate control over their properties to make them highly selective for efficient sensing efficacy. Similarly, integrating chitosan with metal oxide nanostructures improved biosensing selectivity and stability in biological media [10]. Conventional analytical methods are effective, but they have disadvantages due to sluggishness, high cost, and complex action mechanisms for clinical trials. Several electrochemical sensors deliver an improved option because of their high sensitivity, rapid response, and low cost. Standard carbon paste electrodes (CPEs) have limitations of low sensitivity and slow electron transfer rates. So, there is a necessity for the incorporation of modifier materials compatible with the initial functionalities. Although transition metal oxides (such as TiO_2_ and MoO_3_) have been widely explored for excellent energy storage and electrochemical sensing efficacy, they have significant limitations for poor electrical conductivity and instability for electrochemical applications. These limitations have been resolved through new advanced approaches by replacing single metal oxides with hybrid oxide materials. Though another method has been applied to develop more complex materials, such as molybdenum vanadium oxide (M-V-O) by introducing vanadium into MoO_3_ lattices. Such a type of structurally engineered material has superior electronic properties. However, there are major challenges in controlling the size, morphology, and phase transformations of these nanomaterials. But the research demonstrates a paradigm shift towards the broad concept by using metal oxides for designing and architecting multi-metal nanostructures. These materials improve electron transfer rates, surface reactivity, and signal amplification [34]. So, in this regard, Gr-based metal-grafted nanohybrids [35] have emerged as highly promising materials owing to their outstanding physicochemical properties [36–38]. Gr and its derivatives GO, rGO, and pristine Gr offer large surface area, high electrical conductivity, and versatile surface chemistry [39, 40]. This leads to significant improvement in their biomedical capabilities after grafting with metal oxides or noble metals. For example, GO-Au is used for plasmonic sensing and PTT, while rGO-TiO_2_ is effective in ROS generation and imaging [41, 42]. GO-ZnO enables fluorescence-based detection, rGO-Fe_3_O_4_ supports magnetic targeting, Gr-CuO facilitates PDT, and rGO-CeO_2_ offers antioxidant and enzyme-like functionalities [43]. Further have summarized data in Table 1. These nanohybrids not only enhance the sensitivity and specificity of biosensors but also aid in targeted drug delivery and tumor-specific accumulation. The integration of nanotechnology further refines cancer treatment via synergistic platforms. Synthesis methods play a critical role in determining nanostructure morphology, stability, and biological efficacy in cancer treatment [11, 44]. Several studies have shown that synthetic routes such as sol-gel, hydrothermal, and chemical reduction methods control particle size and crystallinity precisely. However, the green synthesis approach using plant or biological extracts improves the eco-friendliness and biocompatibility of NPs though sometimes it may be expensive due to low reproducibility [45, 46]. Recently, green synthesis of magnetic NiCo_2_O_4_/NiO NPs from *Dactylopius coccus* extract and TbFeO_3_/g-C_3_N_4_ nanocomposites using grape juice illustrates the relevance as capping and reducing agents for their significant influences on cytotoxicity outcomes at cancer cell lines because of the overlayer surface and functional groups modulation of nanocomposites. Co_3_O_4_ NPs show inclusive biomedical potential due to their antimicrobial, antioxidant, anticancer, and magnetic properties for their advantageous imaging therapy. *Crataegus microphylla* extract-capped silver NPs (CME@Ag-NPs) are synthesized for a capping agent using *Crataegus microphylla* extract as a green precursor, which demonstrates significant antibacterial activity and anticancer activity against multidrug-resistant strains and cytotoxicity in MCF-7 and AGS cancer cell lines, respectively. So, such metal NPs may extend their properties with Gr for improving their biological efficacy towards cancer theranostics because of synergizing the surface activity and functionalities of hybrid nanomaterials [47, 48]. Experimental methods like sol-gel and hydrothermal routes are efficient because of their important parameters, such as calcination, quality, and molar concentration of chelating ligands and cross-linker, pH buffer, reaction time, and biogenic reducing/capping agents and water-to-feedstock ratio (W/B ratio) help to increase the yield of reactions. A 10% polymeric matrix concentration of Mv-CHT provided optimum dispersibility and electrochemical performance. pH, AgNO_3_ concentration, and reaction temperature may affect the size, morphology, and yield of Mv-CHT-Gr-based nanomaterial synthesis. Furthermore, the concept of architectural synthesis uses a sacrificial template as a novel approach to precisely control size, morphology, and phase of the resulting nano-electrocatalyst. These optimized parameters (such as temperature, pH, concentration, and solvent selectivity) are vital for achieving the desired properties of higher yield and uniform morphology, along with improved functionality like the structural integrity, surface area, stability, and biological activity which can be considered to measure the improved synthetic efficiency successfully. Integration of metals such as Au, Ag, Fe_3_O_4_, TiO_2_, Pt, Pd, Cu, Bi, and ZnO onto graphene’s edges not only enhances electrical conductivity, surface reactivity, and biosensing efficacy but also significantly improves and imparts optical, magnetic, catalytic, and biological properties to the less biologically available pristine graphene. Multifunctionalities not only improve PT and PD therapeutic efficacy but also enable multimodal imaging, biosensing, and controlled drug release. Such an integrated and synergized system creates a multifunctional theranostic platform capable of real-time diagnosis, precise tumor targeting, and simultaneous therapy. To overcome the limitations of conventional cancer treatments, researchers are emphasizing the synergy between metals and graphene for its transformative potential in advancing personalized and minimally invasive oncology. NPs exploit the enhanced permeability and retention (EPR) effect to facilitate passive tumor targeting. Surface engineering allows these particles to evade immune detection, carry therapeutic payloads, and release drugs in a controlled manner. Recent advances of such nanohybrids have illustrated the fundamental mechanisms, innovations, and their significance for cancer theranostics. Metal-grafted Gr-based nanomaterials have emerged as highly versatile single platforms for cancer theranostics, integrating multiple therapeutic and diagnostic functions. The multifaceted advantages of metal-grafted Gr-based nanomaterials make it a promising candidate for addressing major challenges of cancer therapy like low specificity, systemic toxicity, and drug resistance. However, an AI-based study highlights the revolution in breast cancer care through the synergy of AI and metal-grafted Gr-based nanomaterials. AI improves diagnostic accuracy with predictive modeling and improved imaging, while metal-grafted Gr-based nanomaterials ensure precise drug delivery with reduced toxicity. This synergy enables personalized treatments but faces challenges like data quality and model interpretability. Overcoming these challenges through multidisciplinary collaboration promises to significantly improve therapeutic efficacy and clinical outcomes. Gr-metal nanocomposites are especially effective in synergizing multiple therapeutic approaches, including chemotherapy, gene therapy (GT), PTT, PDT, and radiotherapy [49–51]. These therapies yield better tumor targeting, reduce side effects, and improve treatment outcomes compared to monotherapies [52–54]. Brachytherapy, which involves radioactive seed implantation directly into the tumor, limits off-target effects but is restricted to accessible sites. Internal radiotherapy offers a solution by using radionuclide-labeled compounds that localize within tumors, delivering targeted irradiation. Therapeutic radionuclides are categorized based on emission types, primarily alpha (α) and beta-minus (β⁻) particles [55, 56]. α particles, composed of helium nuclei, exhibit high linear energy transfer (LET) and a short penetration depth (28–100 µm), making them ideal for destroying hypoxic tumors [57–59]. They cause direct DNA DSBs that are independent of oxygen levels. β⁻ particles have longer penetration (2–10 mm) and lower LET, acting through ROS-mediated indirect DNA damage [60, 61]. Their ability to affect nearby cells enhances therapeutic reach towards cancer progression, which is driven by the accumulation of genetic mutations that provide malignant cells with an advantageous growth over normal cells [62]. Their resistance to apoptosis, coupled with invasive behavior, promotes metastasis, the spread of cancer to distant sites. In response to early and accurate detection, recent technological advancements have led to the development of Gr-based biosensors. Recent studies have reported significant progress in graphene-assisted phototherapies like gas-facilitated PTT, NIR laser-triggered photo-immunotherapy, and multifunctional hydrogel-based phototherapy [63, 64]. These developments demonstrate the versatility of graphene-based metal nanohybrids as integrated platforms that enable precise, light-triggered cancer therapy by simultaneously supporting multimodal diagnostics and real-time monitoring. PTT combination with nitric oxide gas therapy, PDT, chemotherapy, and immunotherapy has led to innovative multimodal nanoplatforms that enhance therapeutic efficacy by minimizing side effects [65]. Additionally, novel nanostructures such as antibody-conjugated carbon dots and hydrogel composites (AuNPs-CuCCDs@Gel), incorporating copper carbon dots (CuCCDs) and Au, have shown excellent PT and PD antibacterial performance. In particular, graphene-based electrochemical biosensors have shown great promise for oral cancer detection through salivary biomarkers. These sensors offer high sensitivity, specificity, and potential integration with AI and microfluidic platforms for personalized healthcare. Collectively, these advancements highlight that graphene-based systems and emerging phototherapy strategies are converging to drive the next generation of cancer theranostics [66, 67]. These devices integrate with a bio-receptor that specifically identifies cancer biomarkers with a transducer that converts this recognition into a measurable signal. The “seed and soil” theory suggests that metastatic cells colonize microenvironments conducive to their survival, while Ewing’s theory emphasizes anatomical pathways like blood vessels and lymphatics [68, 69]. Immune system surveillance and cellular traits influence metastasis, which remains incompletely understood. Surface functionalization of the transducer is critical to enhancing bio-recognition efficiency and is commonly achieved through covalent bonding, adsorption, or encapsulation techniques. However, it is prone to aggregation and instability in colloidal systems. To mitigate these issues, researchers have explored grafting metals and metal oxides onto Gr to enhance its performance.

**Table 1:** Examples of metal-grafted graphene nanocomposites.

**Serial number**	**Metal-grafted graphene**	**Efficacy**	**Samples**	**Site of action**	**References**
1	Silver-grafted graphene (Ag-Gr)	Enhances conductivity and plasmonic sensitivity for optical biosensing.	Plasma, urine, serum, and stool.	Biofluids or tissues.	[70, 71]
2	Gold-grafted graphene (Au-Gr)	Provides biocompatibility and facilitates biomolecule attachment.	Blood, serum, saliva, urine, cerebrospinal fluid, and tissue biopsies.	Blood vessels, tumor microenvironments, neural tissues, and implant surfaces.	[72]
3	Zinc oxide-graphene (ZnO-Gr)	Offers high electron mobility and photocatalytic activity for electrochemical sensing.	Blood, plasma, urine, saliva, sweat, and tissue extracts.	Tumor tissues, blood vessels, and skin surfaces.	[73, 74]
4	Iron oxide-graphene (Fe_2_O_3_-Gr or Fe_3_O_4_-Gr)	Adds magnetic properties for targeted detection and imaging.	Blood, plasma, serum, saliva, urine, cerebrospinal fluid, and biopsies.	Tumor tissues, lymph nodes, liver, and spleen.	[75]
5	Manganese dioxide-graphene (MnO_2_-Gr)	Improves charge transfer and sensing efficiency.	Blood, plasma, serum, cerebrospinal fluid, saliva, urine, and biopsies.	Tumor tissues, neural tissues, implanted sensor interfaces, and blood vessels.	[76, 77]
6	Titanium dioxide-graphene (TiO_2_-Gr)	Delivers stability and enhanced electron transport for biosensing.	Blood, plasma, serum, saliva, and urine.	Tumor microenvironments, blood vessels, skin surfaces, and implanted medical devices.	[78–80]
7	Cobalt oxide-graphene (Co_3_O_4_-Gr)	Provides strong redox properties for signal enhancement.	Blood, plasma, serum, saliva, biopsies, and urine.	Tumor tissues, neutral tissues, implanted sensor locations, and blood vessels.	[81, 82]
8	Nickel oxide-graphene (NiO-Gr)	Displays high electrocatalytic activity for non-enzymatic sensors.	Blood, plasma, serum, urine, and saliva.	Blood vessels, skin surfaces, and tumor tissues.	[83, 84]
9	Zirconium dioxide-graphene (ZrO_2_-Gr)	Known for chemical stability and effective biomolecule immobilization.	Plasma, blood, urine, serum, tissues, biopsies, and saliva.	Bone surfaces, blood vessels, and tumor tissues.	[85–87]
10	Copper oxide-graphene (CuO-Gr)	Boosts catalytic and conductive properties for fast signal generation.	Urine, serum, tissues, biopsies, plasma, saliva, and blood.	Wound sites, blood vessels, and tumor tissues.	[88–90]

These Gr-based hybrids enhance biosensor performance by increasing sensitivity, providing effective bio-receptor binding surfaces, and supporting multifunctional detection platforms. Their unique physicochemical properties, derived from both Gr and the grafted metal/oxide, enable more accurate, reliable, and efficient cancer diagnostics. By synergizing superior sensing capabilities with enhanced therapeutic delivery systems, these nanohybrids contribute significantly to early diagnosis, improved treatment outcomes, and the reduction of cancer-related mortality.

## Multifaceted carbon nanomaterials for cancer nanomedicine

**Figure 2:**
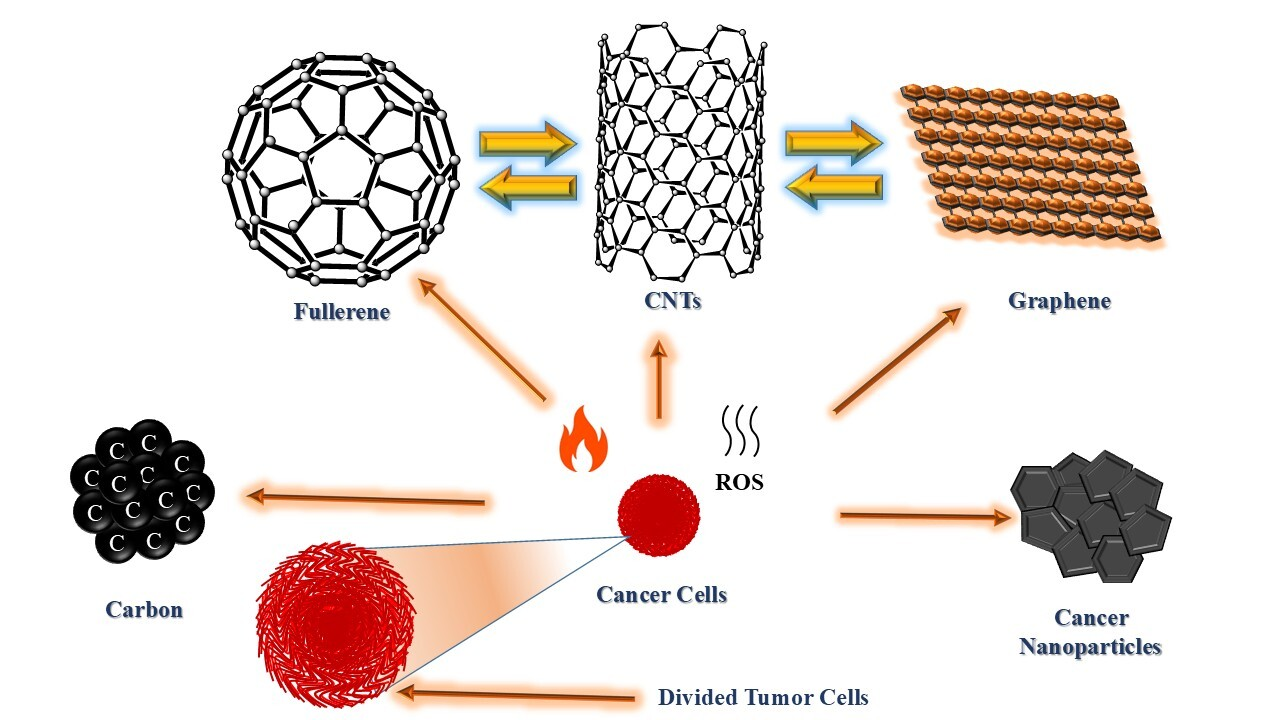
**Carbon nanostructures for therapeutic uses by targeting tumor cells.** CNTs: carbon nanotubes; ROS: reactive oxygen species.

Carbon-based nanomaterials like fullerenes, CNTs, Gr, and Gr-derivatives have gained considerable attention in nanomedicine due to their NIR fluorescence activity, high chemical stability, and ease of functionalization [91–93]. These materials are focused on their role in nanomedicine. Therapeutic applications discussed targeted include chemotherapy, PTT/PDT, and multimodal cancer treatments. Diagnostic efficacies are emphasized in biosensing, biomarker detection, and advanced imaging techniques. In this context, theranostics show dual-function nanoplatforms that combine therapy and diagnosis, including emerging AI-driven precision in oncology. Additionally, biocompatibility and toxicity of nanocomposites are considered as the key challenges and future perspectives for clinical trials. Recent advancements in the radiolabeling of carbon nanomaterials offer potential in cancer therapy extending beyond drug delivery to include targeted tumor destruction with minimal harm to healthy tissues [94, 95]. Their nano size enables conjugation with ligands, nucleic acids, peptides, or antibodies for selective targeting. Additionally, their intrinsic properties, such as ROS and the generation of heat or in situ reactive species, make them promising for direct therapeutic uses. Self-therapeutic nanomaterials have been effectively used to eliminate cancer cells with mechanisms involving ROS production and enzyme growth inhibition. For example, boron-containing materials are used in neutron capture therapy [96, 97]. Fullerenes (C_60_) were discovered by Kroto et al. [98] in 1985. C_60_ and bulkier forms of fullerenes like C_70_ are spherical carbon nanostructures that are encapsulated with metals forming metallofullerenes [99]. These nanostructures can be radiolabeled and functionalized through known organic molecules [100]. CNTs are formed by rolling Gr sheets as single-walled CNTs (SWCNTs) or multi-walled CNTs (MWCNTs). Hence, their large surface area and modifiability make them suitable with agents like polydopamine and polyethylene glycol, enhancing therapeutic efficacy for a long time [101, 102] (Figure 2).

## Multifaceted metal grafted graphene for cancer therapeutics and diagnostics

**Figure 3:**
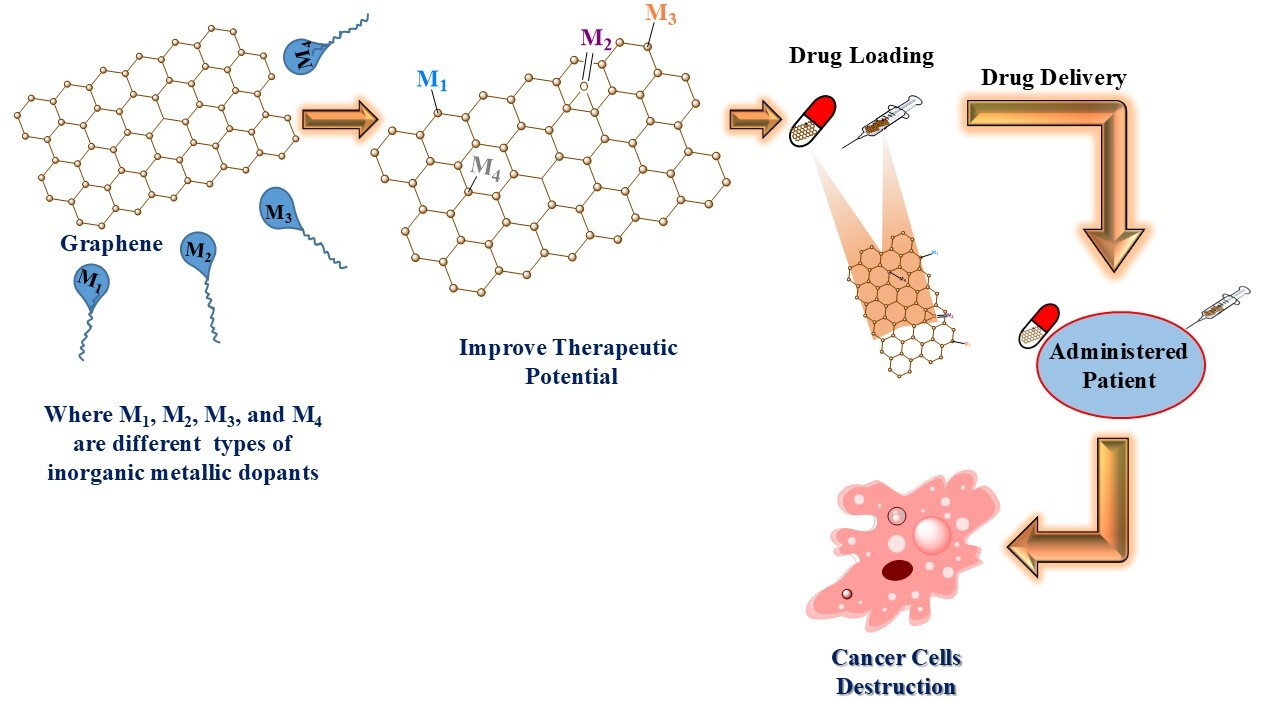
Catalyst-driven graphene functionalization for a smart drug delivery system.

Carbon-based nanostructures such as Gr, GO, rGO, and graphene quantum dots (GQDs) offer exceptional properties for biomedical applications [103–106]. These materials exhibit excellent mechanical strength, thermal stability, electrical conductivity, and tunable optical features, making them ideal for cancer therapy, biosensing, and imaging [107]. Gr is a single-layered hexagonal lattice of carbon atoms, which is synthesized using either top-down or bottom-up methods. Top-down techniques comprise mechanical exfoliation, liquid-phase dispersion, and electrochemical reduction, which break down bulk graphite into a number of single-layered graphene [108–110]. Bottom-up approaches comprise chemical vapor deposition (CVD), epitaxial growth, and molecular synthesis, assembling Gr from smaller chemical units [108, 111]. GO is synthesized through the most effective oxidative process using Hummer and Tour methods. So, the Tour method is considered safer and more eco-friendly [112, 113]. This method introduces oxygen-containing functional groups (hydroxyl, epoxy, carbonyl, and carboxyl) that enhance hydrophilicity and reactivity. However, these functional groups reduce electrical conductivity, which is restored by reducing GO into rGO using thermal, chemical, or electrochemical processes [114]. CVD allows the formation of uniform Gr films but requires sophisticated equipment and may also release toxic by-products. Alternatively, sustainable biosynthesis uses of biological agents like bacteria, fungi, or plant extracts to produce NPs under milder conditions [115, 116]. These biosynthesized nanomaterials are biocompatible and biodegradable, suitable for drug delivery, GT, imaging, and antibacterial applications [117, 118]. Further, inorganic metal grafting onto the surface of Gr also plays a significant role in cancer treatment. Platinum-based drugs (cisplatin, carboplatin, oxaliplatin), ruthenium complexes (NAMI-A, KP1019), and gold agents (auranofin) [119–121] are schematic highlights of an innovative and promising approach for cancer therapy using metal-functionalized Gr nanocomposites. Therapeutic efficacy of Gr is enhanced through the incorporation of metals such as ruthenium (Ru), gold (Au), platinum (Pt), and palladium (Pd), which significantly elevate its biomedical performance via a synergistic effect [122]. In vitro half maximum inhibitory concentrations (IC_50_) studies indicate that the toxicity of Fe_3_O_4_-Gr [123], γ-Fe_2_O_3_-Gr, Au-Gr [124], and Bi-Gr [125] is dose and time-dependent. Studies on HBE, MCF-7, HeLa B, and LNCaP cell lines observed at variable concentrations of 10–200 µg/mL, considering lower concentrations safer for cell viability studies [126]. Au-Gr demonstrates possible selectivity against breast cancer, while Bi-Gr was tested on human liver cancer (HepG2) cell line, which exhibits higher reactivity despite safer consideration of Bi at ~53–88 µg/mL [125]. Pd-Gr and Pt-Gr composites show high reduction in cell viability on LNCaP prostate adenocarcinoma and human ovarian cancer cell lines, respectively, at doses of 10–50 µg/mL [126]. However, a 50% reduction of cell viability and proliferation has been observed at a higher concentration of 100 µg/mL of Pt-Gr [126]. Cell viability studies of ZnO-Gr at three different concentrations of 12.5 µg/mL, 25 µg/mL, 50 µg/mL [127], Ag-Gr at 2 µg/mL [128], and Cu-Gr at 50 μg/mL [129] have been reported with identifying the lower concentrations as safer for the breast cancer cell line MCF-7. In vivo toxicity studies for metal-grafted Gr-based materials are inadequate and often extrapolated from studies on specific Gr-based nanomaterials. The median lethal dose (LD_50_) values for Fe_3_O_4_-Gr and γ-Fe_2_O_3_-Gr are under investigation [130]; while injected Fe_3_O_4_ nanomaterials exhibit an LD_50_ of ~163.6 mg/kg in mice, and death is caused by fast circulation of nanomaterials in the liver, lung, and spleen. However, the associated proteins are denatured. Cell death is observed in the cardiac muscle along with kidney failure. Surface coatings of nanomaterials help mitigate these effects. Intravenously injected Pt NPs have established short-duration safety in rats at therapeutic doses of 10–20 mg/kg without detectable organ damage, while death is caused by the accumulation of Pt nanomaterials in the liver, spleen, kidney, and heart [131]. Au-Gr remains less studied, while Au NPs show size-dependent toxicity on mice, with 8–37 nm being most toxic [132]. Bi-Gr of ~33–38 nm size is usually considered cytotoxic on NRK52E and HepG2 cells, whereas Pd-Gr may induce dose-dependent cytotoxicity [125]. 20 nm ZnO, 8–20 nm Ag, and 23.5 nm Cu NPs characteristically exhibit a higher toxicity (LD_50_) at 5–2,000 mg/kg, 413 mg/kg, and 5,000 mg/kg, respectively [131, 133, 134]. So, the toxicity of NPs depends on their doses and particle size, which causes mice death due to accumulation in the liver, kidney, spleen, and heart. So, there are several future in vivo opportunities that still need to be explored for the toxicity study by grafting of metal NPs with Gr by intravenously administered route to minimize the LD_50_ of NPs. These modifications improve the Gr’s drug-loading capacity, biological compatibility, and targeted delivery efficiency [135]. Once therapeutic agents are loaded, the resulting nanocarriers can be administered through oral or injectable routes, offering flexibility and patient-friendly treatment options. Upon targeting the tumor site, the nanocomposite effectively delivers the drug payload, enabling precise and controlled cancer cell elimination. This advanced system maximizes therapeutic outcomes while minimizing side effects, offering a powerful and efficient strategy for safe, targeted, and personalized cancer treatment [136]. Lab-scale studies and patient use primarily arise from the differences between experimental models and real physiological environments. While in vitro and in vivo studies are typically tested on specific cell lines or animal models. These conditions do not fully replicate the complexity of the human body, including metabolism alteration, immune responses, and other physiological functions. So, the obtained results at the laboratory scale may not directly translate into clinical outcomes. Therefore, there is a need to highlight the extensive preclinical and clinical studies before patient use (Figure 3).

These show promise in targeted drug delivery, PDT, and imaging due to their ROS-generating capability and high surface reactivity. There is a rapidly growing interest among researchers in the use of conjugate graphene-based nanomaterials for cancer treatment and diagnosis (theranostics). However, the graphene field is lacking in research from the oncophysics point of view. The primary application so far has been discovered for drug and gene delivery (73%), followed by PTT (32%), PDT (10%), and imaging (31%). Metal grafted graphene-based nanomaterials focus on these therapeutic techniques to treat the cancerous cells with minimal harm to healthy cells. Its potential lies in revolutionizing medicineʼs ability to diagnose and treat cancer simultaneously. However, oncophysics encompasses numerous onco-techniques, such as fluorescence, electron-beam imaging, and ultrasonography, which can be synergized with one or more therapeutic modalities. The synergize technology of Gr-based nanomaterials and onco-techniques may significantly enhance their opportunity to develop a real revolution in the field of medical therapy if conjugate diagnosis and therapy are simultaneously. Gr-based nanomaterials have achieved improved targeting ability, reduced side effects, and controlled release profiles [137]. GQDs, below 100 nm, possess strong fluorescence and a large surface area [138, 139]. Their surface can be easily functionalized with biomolecules for selective recognition of cancer biomarkers. GQDs enable sensitive detection, early diagnosis, and therapeutic monitoring, positioning them as cutting-edge tools for on-site cancer diagnostics [140]. So, metal-grafted Gr is expected to enable highly reactive cancer treatments (Table 2), as well as improved diagnostic tools for early detection and continuous monitoring, ultimately leading to better therapeutic outcomes.

### Metal-grafted graphene as a nanocarrier for targeted chemotherapy

**Figure 4:**
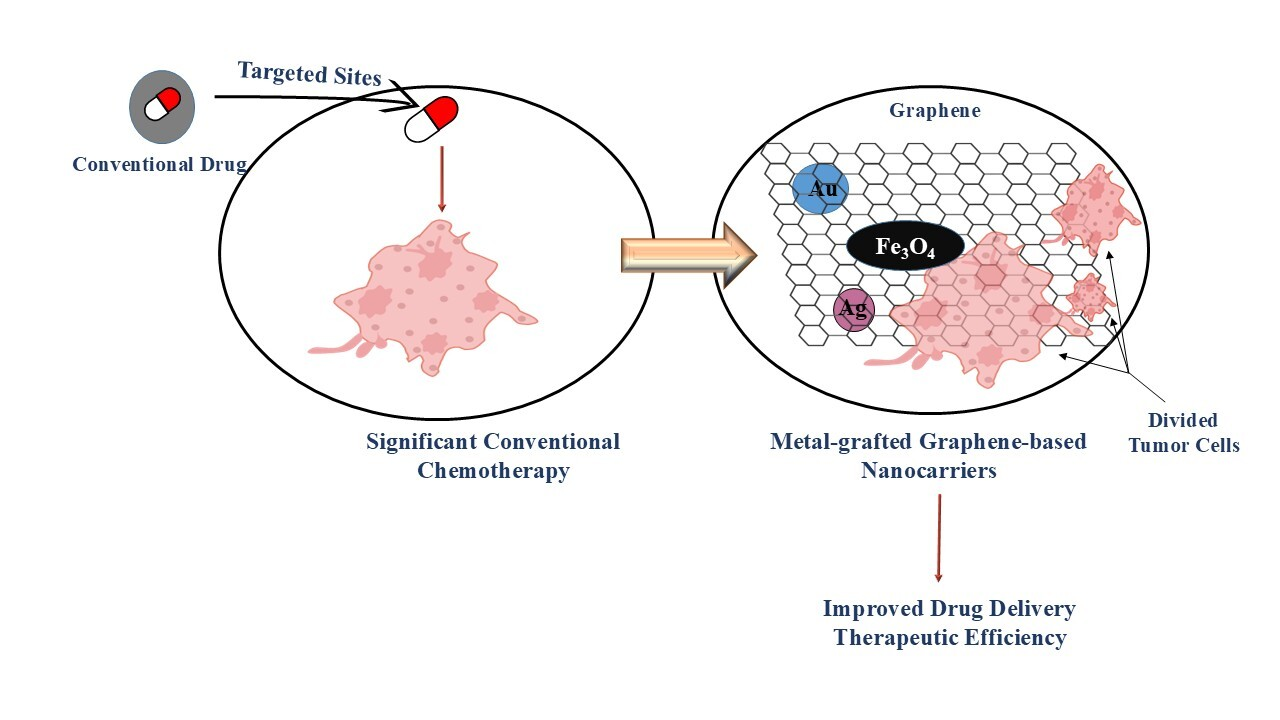
Improved drug targeting and tumor reduction using metal-grafted graphene.

The origin of chemotherapy traces back to the discovery that sulfur mustard gas irreversibly damaged bone marrow and produced unnecessary cell growth [141]. This observation raised concern for researchers to investigate similar types of compounds for targeting fast-growing cancerous cells. So, this effort led to the development of non-cytotoxic drugs that remain vital for cancer treatment. These medications are primarily intended to attack rapidly multiplying cancerous cells. However, it also harms healthy cells, which may cause to divide cells of hair follicles, bone marrow, and gastrointestinal tract resulting in side effects like fatigue, nausea, hair loss, and infertility [142] (Figure 4).

Alkylating agents are one of the earliest chemotherapeutic classes, disrupting the DNA into a number of divided cells, resulting in cell death [143, 144]. Another class of chemotherapeutic agents is microtubule stabilizers that interfere with cell division by preventing microtubule breakdown [145]. Such a type of supportive therapy helps manage these side effects, and most symptoms lessen after the end of treatment. Despite side effects of chemotherapy, it remains highly effective against testicular cancer, survival rate of up to 95%. So, nanotechnology-based drug delivery systems have been developed to make efficient, targeted, and reduced side effects therapy. Hyaluronic acid (HA)-hybrid NPs are targeted to breast cancerous stem cells through cluster of differentiation 44 (CD44) receptor interaction [146, 147]. These NPs are synthesized by binding of lipids with poly(lactide-co-glycolic acid) (PLGA), resulting in superior cellular uptake [148, 149]. Fluorescence microscopy and confocal imaging confirmed that HA-hybrid NPs had a 72% higher uptake than PLGA NPs and showed specificity due to CD44 binding [25, 150, 151]. Gr doped with metals like Au, Ag, and Fe_3_O_4_ enhances drug delivery and cancer therapeutic efficiency [152]. Au-Gr improves PTT by converting NIR light into heat, selectively destroying tumor cells [153–155]. AgNPs grafted onto Gr possess cytocompatibility and antimicrobial properties, increasing the effectiveness of chemotherapy while reducing infection risks during treatment [156–158]. Similarly, Fe_3_O_4_-Gr composites offer dual functionality as drug carriers and magnetic agents for targeted therapy and imaging [159–162]. The integration of chemotherapy with cutting-edge nanotechnology and metal-grafted Gr-based materials holds a significant role for advancing personalized cancer treatment [163, 164]. Approaching studies are likely to emphasize the creation of versatile nanocarriers that have the capability of delivering therapeutic agents with exceptional precision while simultaneously enabling real-time diagnosis and treatment monitoring. Ongoing investigations with Gr composites and metal-integrated nanostructures may lead to the development of intelligent environment-responsive drug delivery platforms tailored to the unique characteristics of tumor tissues [165]. Additionally, optimizing biocompatibility, minimizing adverse systemic effects, and ensuring scalable, cost-effective production will be essential for their transition into clinical settings. As these multidisciplinary innovations progress, they are poised to redefine cancer management by reducing harmful side effects, addressing drug resistance, and enhancing overall patient outcomes and longevity.

### Metal-grafted graphene from targeted delivery to precision diagnostics

Cancer endures to claim millions of lives annually, making it one of the deadliest diseases worldwide. Over the past two decades, cancer research has grown rapidly and particularly with the emergence of NPs and their innovative applications. Numerous cancer types exist, including breast, lung, colorectal, and prostate cancers [166]. According to the American Cancer Society, breast, lung (non-small cell), prostate, colorectal, and melanoma are the most prevalent across U.S. states recently [167]. Metal-grafted Gr nanocomposites exhibit exceptional biomedical sensing and therapeutic applications. These are typically fabricated through techniques like electrochemical deposition, chemical reduction, sonication, microwave-assisted methods, and photo-induced reduction [168, 169]. A popular route involves the simultaneous reduction of metal precursors and oxidation of Gr, often stabilized using surfactants or co-ligands to prevent NP aggregation and improve dispersion [170]. In breast cancer therapy, conventional chemotherapeutics have progressed, but nonspecific toxicity toward healthy cells remains a significant challenge. To overcome this, cyclodextrin (CD)-functionalized polyurethane (PU)-based magnetic NPs were synthesized [171]. Initially, a dipodal silane carbamate-functionalized alkoxysilane (ECA) was formed by reacting 3-aminopropyltriethoxysilane and γ-glycidoxypropyltrimethoxysilane in dimethyl sulfoxide under nitrogen to avoid moisture [172]. Fe_3_O_4_ NPs were prepared via the coprecipitation method using ferric and ferrous chlorides with ammonium hydroxide at pH 11 [173–175]. Then, these were coated with ECA and combined with PU to form Fe_3_O_4_-ECA-PU-CD nanocomposites [171, 172]. The resulting system has been demonstrated to enhance targeted and reduced off-target toxicity in breast cancer therapy. Furthermore, arginine-glycine-aspartic acid (RGD)-doxorubicin (DOX) loaded solid lipid NPs (SLNPs) showed 5.58-fold higher plasma exposure, indicating improved drug delivery efficiency [25, 176, 177]. Among plasmonic NPs, AuNPs are widely used due to their resistance to oxidation with a strong surface reactivity and biocompatibility [178]. So, rGO-Au composites were synthesized via a hydrothermal method, which were used in electrochemical sensors for detecting prostate-specific antigen (PSA) with a detection limit of 50 pg/mL [179–181]. Additionally, doping of Fe_3_O_4_ and ZnO with Gr enhances both magnetic targeting and ROS-mediated cancer cell apoptosis [182, 183]. These multifunctional nanocomposites serve as promising platforms for drug delivery, imaging, and PTT or oxidative therapy. Thereby, these improve therapeutic precision and efficacy. However, an AI-based study highlights the revolution in breast cancer care through the synergy of AI and metal-grafted Gr-based nanomaterials. AI improves diagnostic accuracy with predictive modeling and improved imaging, while metal-grafted Gr-based nanomaterials ensure precise drug delivery with reduced toxicity [184]. This synergy enables personalized treatments, but it has challenges like data quality and model interpretability. Overcoming these challenges through multidisciplinary collaboration promises to significantly improve therapeutic efficacy and clinical outcomes. Researchers are focusing on the development of intelligent stimuli-responsive nanomaterials that are capable of performing real-time tumor detection, monitoring, and therapy [185]. Functionalization of Gr-based nanostructures with targeting moieties and immunomodulatory agents offers promising avenues for advancing individualized cancer care. Continued efforts to enhance their biocompatibility, minimize adverse effects, and ensure reproducibility are critical for clinical translation. Regulatory validation and extensive clinical testing will be essentially required to transition these advanced nanotherapeutics from laboratory research to standard medical applications, ultimately improving patient outcomes and survival rates.

### Metal-grafted graphene for cancer theranostics and biosensing

The integration of metallic elements onto Gr structures substantially elevates their performance across diverse domains such as biosensing, energy storage, and electronic devices. Also, these composites substantially enhance the efficacy of cancer imaging and treatment strategies. Gr integrated with iron oxide improves MRI precision diagnosis, while Au nanorods on Gr surface increase photoacoustic responses [186]. Au and Bi-doped Gr enhance CT scan resolution, while the ZnO and CuO onto the Gr surface facilitate fluorescence-based tracking of cancer cells [178, 187, 188]. These nanocarriers are functionalized with specific ligands, such as antibodies and folic acid (FA), to achieve targeted drug delivery to tumor sites in therapeutic applications [189, 190]. Au- and Ag-coated Gr absorbs near infrared light and converts it into localized heat for cancer cell ablation (removal of dead tissues) in PTT [155, 191]. Also, metal oxides on the Gr surface generate ROS light exposure, leading to cancer cell apoptosis in PDT [192]. These multifunctional systems also enable dual action approaches by combining drug release and light-triggered therapies, thereby enhancing treatment precision, reducing toxicity, and improving therapeutic outcomes.

Metal-grafted Gr frameworks substantially boost electrochemical biosensing efficacy by optimizing charge transfer and catalytic performance [193]. This advancement facilitates recognition of highly sensitive markers of cancer targets like proteins [PSA, human epidermal growth factor receptor 2 (HER2), and cancer antigen 125 (CA-125)], genetic materials (DNA and miRNA), and extracellular components such as exosomes and circulating tumor cells [194, 195]. Au-GO hybrid sensor equipped with aptamers can detect PSA at exceptionally low (femtomolar) concentrations [196]. Au-Gr strengthens surface plasmon effects, thereby enabling accurate label-free detection for optical biosensing efficacy [197]. Light-sensitive metal-grafted Gr improves fluorescence detection precision [198]. Additionally, Ag-Gr nanocomposites show sensing ability for enzymes to improve colorimetric assays that visually identify cancer biomarkers [197]. Furthermore, metal-grafted Gr nanohybrid nanostructures are being integrated as rapid diagnostic tools offering immediate, non-invasive, and on-site monitoring of cancer markers, which is an essential feature for timely diagnosis and therapeutic evaluation [199]. Metal-grafted Gr provides dual functionality to enable both therapeutic and diagnostic capabilities within a single platform as an ideal configuration for advanced cancer theranostics. Their extensive surface area supports the effective attachment of biomolecules such as enzymes, DNA, or antibodies, which is crucial for selective targeting and biosensing ability [200]. Chemical surface properties of Gr are readily modifiable to improve its biocompatibility. These nanohybrids also exhibit responsiveness to external stimuli during pH and temperature variations, light exposure, thereby facilitating controlled therapeutic delivery and adaptable diagnostic responses [201].

Metal-grafted Gr nanocomposites demonstrate significant potential in cancer diagnosis and treatment, but several key challenges persist that require attention. Issues such as potential long-term toxicity and limited biocompatibility of certain formulations remain a concern [202]. Furthermore, consistent synthesis with higher stability and reproducibility presents difficulties in large-scale production and clinical approval, which endures a significant hurdle [203]. However, there is a strong authoritative process for advanced multifunctional platforms of precisely controlled drug delivery. Crucially, thorough in vivo evaluations and rigorous clinical trials will be essentially required to facilitate their safe and successful integration into real-world medical applications. Future studies should prioritize the development of environmentally sustainable and scalable manufacturing approaches.

### Biocompatible metal-grafted graphene for targeted cancer drug delivery

Conventional chemotherapy is often constrained by widespread toxicity, suboptimal target sites, and the emergence of resistance. To overcome these challenges, metal-grafted Gr has analysed advanced solutions for site-specific delivery as anticancer agents. Among these, Gr-based nanostructures have gained significant attention owing to their exceptional physical and chemical properties [204]. The incorporation of biocompatible metals onto the Gr surface further expands its therapeutic potential by drug loading efficiency, which enables controlled drug release, supporting imaging applications, and facilitating selective accumulation in tumor tissues [205].

Gr derivatives such as GO, rGO, and GQDs are well-suited for drug delivery owing to their extensive surface area, ability to interact with aromatic compounds, tunable surface chemistry, and robust mechanical properties [92]. Pristine Gr has less biocompatibility, while it has numerous reactive groups for its surface modifications. However, oxidation, reduction, and metal-grafting are performed to augment its biomedical functionality [206]. Metals are grafted onto the Gr surface via chemical reduction, electrochemical methods, or green synthesis using natural agents [207]. Metals incorporation confers distinct therapeutic advantages. Au mediates PTT, imaging diagnostic, which also enhances biocompatibility along with antimicrobial properties and ROS generation. Pt facilitates dual-action chemotherapy. Fe_3_O_4_ enables magnetic and MRI-based imaging. ZnO promotes pH-triggered drug release and induces cell apoptosis. Cu aids PDT and inhibits tumor angiogenesis [43, 161]. These functional enhancements confirm metal-grafted Gr as a versatile platform for targeted and multimodal cancer treatment.

Metal-functionalized Gr platforms support both passive target through the EPR effect and active target via ligands such as FA, HA, antibodies, aptamers, and peptides that specifically recognize receptors of cancer cells, thereby improving delivery precision [208]. These nanocarriers exhibit excellent drug-loading capacity and enable stimulus-responsive drug release triggered by acidic pH, thermal energy (photothermal effect), redox environments, or external magnetic fields, allowing for localized therapy with minimal adverse effects [209].

Integrating chemotherapy with additional treatment modalities like PTT (utilizing Au or Cu), PDT (with ZnO or CuO), or magnetically assisted therapy significantly improves therapeutic efficacy, which promotes cancer cell apoptosis and mitigates drug resistance [210]. Experimental results demonstrate biocompatibility with normal cells, efficient uptake by tumor cells, prolonged and controlled drug release, substantial tumor growth inhibition, and support for non-invasive imaging techniques. Notable metal includes Au-GO-DOX and Gr-Fe_3_O_4_-FA-Cisplatin systems [211].

Realizing the future necessitates clinical translation of these nanoplatforms make efforts to focus on sustainable and scalable production using natural templates or plant-derived materials. This leads to the development of advanced multifunctional systems enabling controlled drug delivery, imaging treatment, and comprehensive in vivo testing across diverse cancer models. For this, the implementation of regulatory-compliant strategies for safety, toxicity, and pharmacokinetic assessment is essentially required [212].

### Metal-grafted graphene precision oncology for smart cancer detection and therapy

Inorganic metal-grafted Gr nanocomposites have emerged as multifunctional platforms in the field of precision oncology, which offer dual functions for cancer detection as well as treatment within a single engineered nanosystem [53]. Integration of metals such as Au, Ag, Pd, and Fe_3_O_4_ or γ-Fe_2_O_3_ onto Gr shows high electrical conductivity, large surface area, and functional tunability with the unique optical, magnetic, and catalytic properties [213, 214]. Au-Gr and Ag-Gr nanocomposites enable ultra-sensitive detection of cancer biomarkers through chemical sensing mechanisms that enhance optical and electrochemical signals [215, 216]. Also, catalysts such as Pt and Pd improve signal transduction in electrochemical assays [217]. Additionally, superparamagnetic metal oxide Fe_3_O_4_ NPs serve as MRI contrasting agents, which facilitate early tumor localization [218]. Upon successful identification and accumulation in malignant tissues, the homogeneous and heterogeneous metal-grafted Gr platform can initiate targeted therapy through photothermal ablation, magnetic hyperthermia, or stimuli-responsive drug release, triggered by environmental prompts such as pH, redox gradients, or external fields [219]. Functionalization with ligands like FA or monoclonal antibodies enhances tumor selectivity to ensure precise delivery and minimize systemic toxicity [220]. Furthermore, real-time imaging efficacy of nanostructures allows for dynamic treatment monitoring, which enables adaptive therapeutic strategies. This convergence of sensitive diagnostics and localized therapy within a single system exemplifies the core principles of cancer theranostics, paving the way for more effective, tailored, and minimally invasive clinical interventions.

**Table 2:** Comparison between Metal-grafted graphene as a nanocarrier for targeted chemotherapy and Metal-grafted graphene precision oncology for smart cancer detection and therapy.

**Serial number**	**Metal-grafted graphene**	**Application in cancer theranostics**	**References**
1	Au-Gr	Chemotherapeutic drugs	[155, 156, 159]
2	Fe_3_O_4_-Gr	Detecting biomarkers (PSA)	[178, 183, 187, 188]
3	Ag-Gr	Imaging (CT) biosensing	[43, 156, 161]
4	ZnO-Gr	Biocompatible non-toxic nanocarrier	[161, 182, 188]
5	Pd-Gr	Ultra-sensitive and real-time monitoring	[212, 216]
6	Bi-Gr	MRI	[178, 187, 188]
7	Cu-Gr	Photodynamic	[209]
8	γ-Fe_2_O_4_-Gr	Photothermal	[213]
9	Pt-Gr	Dual-action chemotherapy	[216, 217]

PSA: prostate-specific antigen; MRI: magnetic resonance imaging.

## Conclusions

Metal-grafted graphene nanohybrids synergize progression in cancer theranostics by diagnostic precision with therapeutic efficacy. Several mono-metallic graphene-based nanomaterials, such as Fe_3_O_4_-Gr, γ-Fe_2_O_3_-Gr, Au-Gr, Bi-Gr, Pd-Gr, Pt-Gr, ZnO-Gr, and Cu-Gr, have been studied for their cancer theranostics efficacy, but many are still being explored. However, new opportunities are emerging for the synthesis of bimetallic/trimetallic graphene-based nanomaterials for pursuing their unexplained efficacy. Additionally, these nanocomposites are being explored for in vitro and in vivo toxicity testing to justify their cancer theranostics efficacy. Mono-metallic graphene-based nanomaterials have shown positive in vitro anticancer responses with measured IC_50_ values at lower concentrations. However, in most cases, in vivo assessments with measured LD_50_ cause death of the organisms even after the nanomaterials have been successfully delivered into the cells. These nanohybrids offer multiple theranostic capabilities, including real-time biosensing, targeted drug delivery, multimodal imaging, and therapeutic modalities such as PT, PD, and immunotherapy. Their large surface area, tunable electronic properties, and enhanced catalytic or plasmonic activity make them strong candidates for next-generation nanomedicine as minimally invasive and personalized cancer treatments. Environment-friendly biogenic synthetic methods further strengthen their ecological and clinical applicability. Future studies must focus on a systematic in vivo analysis of biodistribution and clearance, the development of scalable and eco-friendly synthetic methods, and the integration of these nanohybrids with AI, microfluidics, and wearable devices for real-time monitoring and adaptive therapies. AI and metal-grafted graphene nanomaterials are synergistically transforming breast cancer therapy by enhancing diagnostic accuracy, enabling personalized treatment, and supporting targeted drug delivery with reduced toxicity and real-time monitoring. While challenges in data accessibility, manufacturing scalability, and lengthy clinical trials persist, a multidisciplinary, collaborative approach is essential for advancing oncology therapy. Furthermore, improved tumor cell selectivity through functionalization and targeted drug release, along with synergistic use with immunotherapy, can further enhance efficacy, reduce systemic toxicity, and decrease the overall patient burden.

## Abbreviations

CD: cyclodextrin
CD44: cluster of differentiation 44
CNTs: carbon nanotubes
CVD: chemical vapor deposition
DOX: doxorubicin
DSBs: double-strand breaks
EBRT: external beam radiotherapy
ECA: carbamate-functionalized alkoxysilane
EPR: enhanced permeability and retention
FA: folic acid
GO: graphene oxide
GQDs: graphene quantum dots
GT: gene therapy
HA: hyaluronic acid
HepG2: human liver cancer
IC_50_: half maximum inhibitory concentrations
LD_50_: median lethal dose
LET: linear energy transfer
MRI: magnetic resonance imaging
Mv-CHT: V_3.6_Mo_2.6_O_16_-chitosan
NIR: near-infrared
NPs: nanoparticles
PD: photodynamic
PDT: photodynamic therapy
PLGA: poly(lactic-co-glycolic acid)
PSA: prostate-specific antigen
PT: photothermal
PTT: photothermal therapy
PU: polyurethane
rGO: reduced graphene oxide
ROS: reactive oxygen species

## Declarations

### Copyright

© The Author(s) 2025.
